# Preparation and Catalytic Activity of Carbon Nanofibers Anchored Metallophthalocyanine in Decomposing Acid Orange 7

**DOI:** 10.3390/ma7021370

**Published:** 2014-02-20

**Authors:** Baocheng Zhou, Wenxing Chen

**Affiliations:** 1 Department of Chemistry, Zhejiang Sci-Tech University, Hangzhou 310018, Zhejiang, China; E-Mail: zhoubc1982@163.com; 2 Key Laboratory of Advanced Textile Materials and Manufacturing Technology, Ministry of Education of China, Zhejiang Sci-Tech University, Hangzhou 310018, Zhejiang, China

**Keywords:** carbon nanofibers (CNFs), cobalt tetracarboxylphthalocyanine (CoTCPc), Acid Orange 7 (AO7), catalytic decoloration

## Abstract

Amine-modified CNFs (AN-CNFs) were first obtained through the Billups reaction from carbon nanofibers (CNFs), and were used as supports of cobalt tetracarboxylphthalocyanine (CoTCPc) for the catalytic oxidation of Acid Orange 7 (AO7) in the CoTCPc-AN-CNFs/H_2_O_2_ system. CNFs, AN-CNFs and CoTCPc-AN-CNFs were characterized by X-ray photoelectron spectroscopy, thermogravimetric analysis, transmission electron microscopy and N_2_ adsorption-desorption. The oxidative decoloration of AO7 in the presence of CoTCPcNa-AN-CNFs and H_2_O_2_ was investigated by UV-Vis absorption spectra. The results showed that AO7 was oxidized efficiently in the CoTCPcNa-AN-CNFs /H_2_O_2_ system. The benzene ring was first introduced between CNFs and MPcs. However, its catalytic efficiency and electronic properties would not weaken. New catalytic mechanism may display in this CoTCPcNa-AN-CNFs /H_2_O_2_ system.

## 1. Introduction

Industrial development is pervasively connected with the disposal of large numbers of various toxic pollutants including dyes. Dyes are present in the wastewater streams of many industrial sectors such as, dyeing, textile, tannery and the paint industry [1,2]. Azo dyes represent the largest class of textile dyes in industrial use, accounting for 50% of all commercial dyes [3]. They are characterized by nitrogen-nitrogen double bonds [4]. Many of the conventional treatment technologies for dye removal have been investigated intensively such as chemical coagulation or flocculation combined with flotation and filtration, membrane filtration, oxidation, and photo-degradation processes [5,6]. The adsorption process provides an attractive method for the treatment of textile effluent especially if the adsorbent is inexpensive and readily available [7]. Carbon nanofibers (CNFs) (40–100 nm) with their extraordinary thermal, mechanical, and electrical properties, have gained wide-spread attention in the material research community in recent years [8,9]. CNFs are a kind of highly porous material with high specific surface area and large pore volume, which can facilitate the adsorption of organic chemicals. CNFs are extremely promising as supports for heterogeneous metal catalysts for organic synthesis and fuel cell application [10–12]. Some studies have indicated the introduction of CNFs can provide effective long-range electron transfer in electrodes, thereby resulting in improve catalyst utilization and lower catalyst loading [11]. In additional, Meteallophthalocyanines (MPcs) have received increasing attention in some catalytic oxidation systems using H_2_O_2_ as oxidant. Aromatic compounds such as phenols and dyes could be oxidized efficiently in the presence of MPcs molecular catalysts [13–16] or supported MPcs catalysts [17,18].

**Scheme 1.**
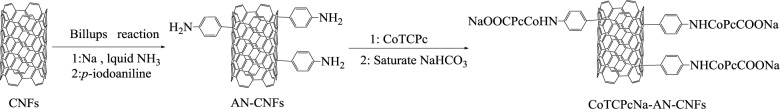
Functionalization of CNFs to produce AN-CNFs and CoTCPcNa-AN-CNFs through the Billups reaction.

Recently, we have reported that supported MPcs can degrade organic pollutants effectively in the presence of H_2_O_2_, and found that supported MPcs are more active and selective than unsupported ones [19–21]. When multiwalled carbon nanotubes (MWCNTs) were used as a support, it was suggested that some π-conjugated dyes can approach CNTs to improve the catalytic activity of MPcs, since every carbon atom of CNTs contributes one electron to establish π-π electrostatic interactions [21–24]. Although CNFs do not mirror the properties of CNTs, they still provide a unique combination of mechanical, electrical and thermal properties to materials. CNFs are more economical and offer an inexpensive way to exploit the physical properties of CNFs. In line with our research on the relationship between CNTs and MPcs, CNFs were chosen as a support for MPcs due to their similarity with CNTs. Here, we focused on CoTCPc immobilized covalently on CNFs modified by the Billups reaction [25], which generally possess greater adsorption capacity, to obtain a heterogeneous catalyst (CoTCPcNa-AN-CNFs) (Scheme 1). AO7 was chosen as a model of the hydrosoluble phenylazonaphthol dyes due to its wide application and resistance to biological degradation. The dye decolorization of AO7 in the presence of CoTCPcNa-AN-CNFs and H_2_O_2_ was investigated by UV-vis absorption spectra at the maximum absorption wavelength for AO7 (λ_max_ = 484 nm). The effects of catalyst loading, temperature, pH values and salt concentration were studied in this paper. Besides, the reaction mechanism was also investigated. Importantly, we further investigated whether the introduction of whether the benzene ring between CNFs and MPcs could affect the electronic properties of CNFs. To our best knowledge, the heterogeneous catalyst (CoTCPcNa-AN-CNFs) and the system of CoTCPcNa-AN-CNFs/H_2_O_2_ have not yet been reported.

## 2. Results and Discussion

### 2.1. Characterization of CoTCPcNa-AN-CNFs

**Figure 1.**
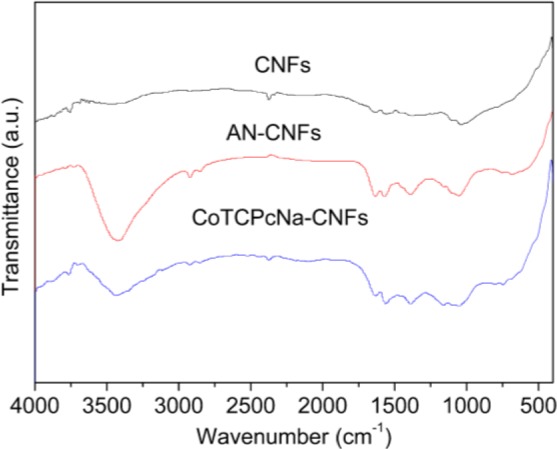
Fourier transform infrared spectroscopy (FT-IR) for carbon nanofibers (CNFs), 4-aminophenyl carbon nanofibers (AN-CNFs), CoTCPcNa-CNFs.

The bonding forms of CoTCPcNa-AN-CNFs were characterized using XPS and FTIR, and thermal stability was determined by TGA. FTIR and XPS experiments were both used to prove the formation of covalent bonding between CoTCPc and AN-CNFs. FTIR spectra were used to characterize the functionalization of amid linkage (Figure 1). In AN-CNFs, the bands centered at 3400, 1600, 1200 cm^−1^ are attributed to asymmetric stretching, symmetric stretching of –NH_2_. For CoTCPcNa-CNFs, the strength of 3400 cm^−1^ shown some weaken, and the peak at 1682 and 1545 cm^−1^ were assigned to amide and amide linkage between CoTCPc and AN-CNFs [21]. FTIR spectra indicated that CoTAPc was immobilized on the CNFs via amide linkage.

**Figure 2.**
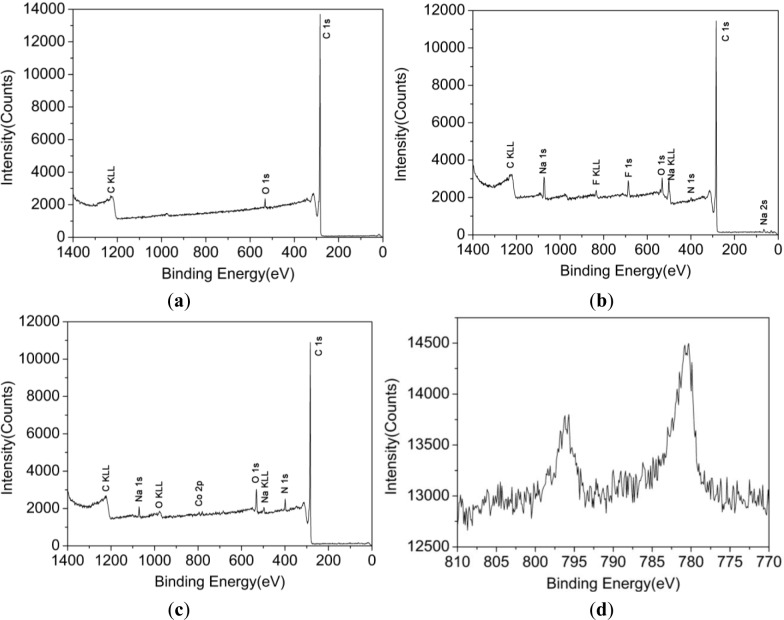
(**a**) XPS spectra of CNFs; (**b**) XPS spectra of AN-CNFs; (**c**) XPS spectra of CoTCPcNa-CNFs; (**d**) XPS spectra of CoTCPcNa-CNFs.

The XPS spectra from a wide scan of CNFs, AN-CNFs and CoTCPcNa-AN-CNFs were shown in Figure 2 respectively.

As shown in Figure 2b, the new band of nitrogen was detected, suggesting that 4-aminophenyl bonded with CNFs. Besides, new bands of sodium and fluorine were also detected, showing that some undetermined reactions took place during the Billups reaction. A marked increase in oxygen and nitrogen was detected in Figure 2c, owing to the amidation reaction used for bonding CoTCPc and AN-CNFs. Moreover, the new band of cobalt was detected, and the peaks at 795.62 eV and 780.37 eV were attributed to Co 2p^1^/2 and Co 2p^3^/2 respectively [21] (Figure 1d). According the FTIR and XPS spectrum, we concluded that CoTCPc was immobilized on AN-CNFs via amide linkage.

**Figure 3.**
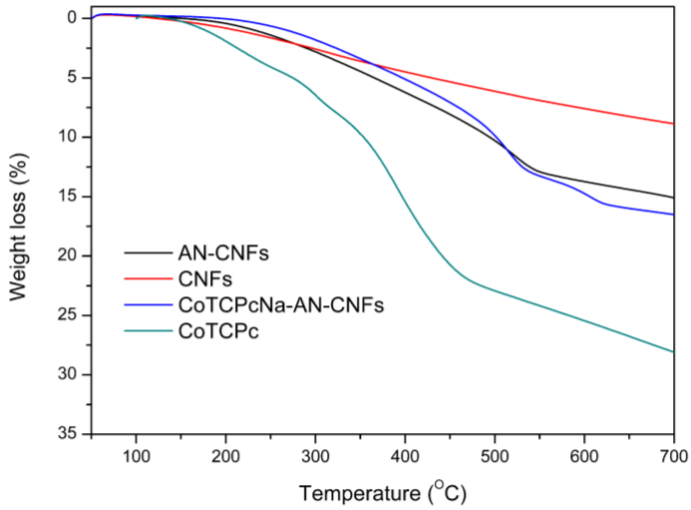
Dynamic thermogravimetric analytical curves of CNFs, AN-CNFs, CoTCPcNa-AN-CNFs and CoTCPc.

The thermal stabilities of CNFs, CoTCPc, AN-CNFs and CoTCPcNa-AN-CNFs were evaluated by TGA (Figure 3). CNFs have excellent thermal stability in the range 50–700 °C. The decomposition of CoTCPc started at 415 °C and when the temperature increased to 500 °C, the main residues were some inorganic cobalt-containing compounds. When CNFs were modified by the Billups reaction, the decomposition temperature of AN-CNFs was about 520 °C. The curve of the weight loss of CoTCPc, AN-CNFs and CoTCPcNa-AN-CNFs in the temperature between 400 and 500 °C were very similar with each other, indicating that CoTCPc was fixed on the CNFs in some aspect.

**Figure 4.**
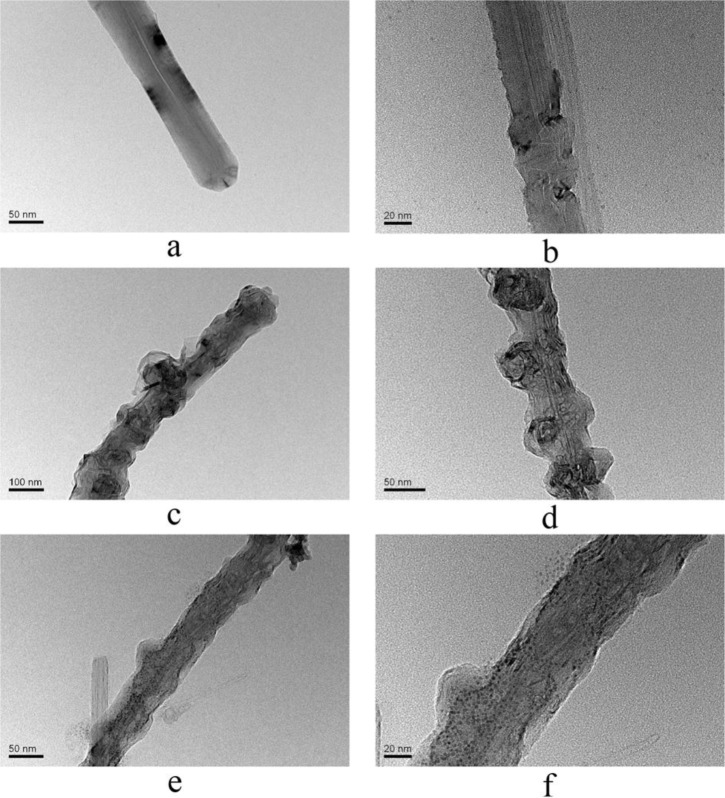
TEM image of the CNFs, AN-CNFs and CoTCPcNa-AN-CNFs. (**a**) CNFs at 50 nm; (**b**) CNFs at 20 nm; (**c**) AN-CNFs at 100 nm; (**d**) AN-CNFs at 50 nm; (**e**) CoTCPcNa-AN-CNFs at 50 nm; (**f**) CoTCPcNa-AN-CNFs at 20 nm.

TEM was employed to investigate the morphology of catalyst. Figure 4 shows the typical TEM images of CNFs, AN-CNFs and CoTCPcNa-AN-CNFs. Figure 4a shows that CNFs have similar structure with multiwalled carbon nanotubes (MWCNTs), and have sandwich structure as shown in Figure 4b. Before functionalization, the CNFs surfaces are smooth with no defects. However, some drawbacks were detected and the CNFs surface became irregular after modified by the Billups reaction (Figure 4c,d). Figure 4e,d shown TEM images of CoTCPcNa-AN-CNFs, some unconfirmed particles were found in the surface of CNFs, owing to the fact that the MPcs molecules were aggregated and displayed dimer structure in solution [26]. Therefore, we inferred that CoTCPc has fixed on the CNFs.

**Figure 5.**
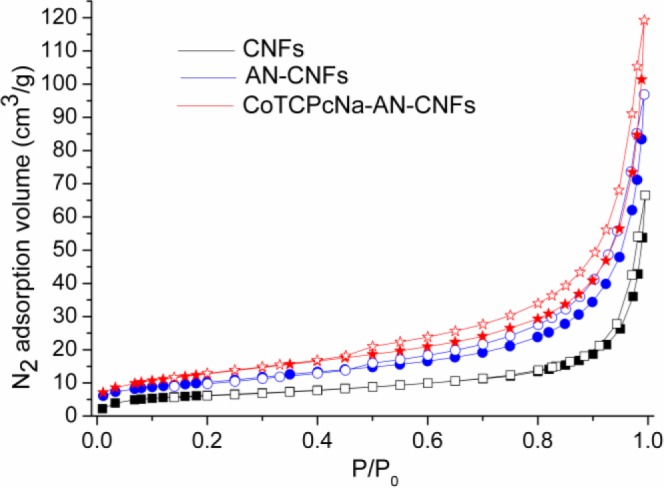
N_2_ adsorption-desorption isotherms.

Figure 5 shows the N_2_ adsorption-desorption isotherms of CNFs, AN-CNFs and CoTCPcNa-AN-CNFs. The BET surface areas of CNFs, AN-CNFs and CoTCPcNa-AN-CNFs are 22.6439, 36.4363, 46.6347 m^2^·g^−1^, respectively. The small increase of nitrogen adsorption volume suggests the disorder of CNFs was litter changed after the Billups reaction [25].

### 2.2. Oxidative Decoloration of AO7

**Figure 6.**
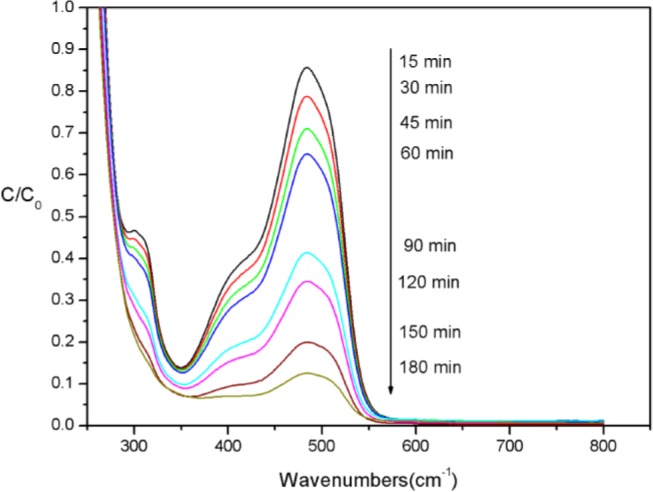
UV-vis spectra changes with reaction time [*C*(AO7) = 5 × 10^−5^ mol/L, *C*(CoTCPcNa-AN-CNFs) = 0.333 g/L, *C*(H_2_O_2_) = 4.85 × 10^−2^ mol/L, 25 °C].

AO7 was selected as the model of azo dyes in order to investigate the catalytic performance of CoTCPcNa-AN-CNFs. Because of the limited oxidation power of H_2_O_2_ (*E*^0^ =1.78 V), H_2_O_2_ is not powerful enough when used alone and cannot oxidize AO7 directly [27]. To clarify the changes in molecular and structural characteristics of AO7 as a result of oxidation via CoTCPcNa-AN-CNFs/H_2_O_2_ process, representative UV-visible spectra changes of the dye solution as a function of reaction time were observed and the corresponding spectra are shown Figure 6. Before the oxidation, the absorption spectrum of AO7 was characterized by one main band in the visible region (λ_abs_ = 484 nm), and the other one in the ultraviolet region located at 310 nm, respectively. The peak at 310 nm was associated with “benzene-like” structure in the molecule, and the 484 nm band is originated from an extended chromophore (nitrogen -nitrogen double bonds), comprising both aromatic rings connected through the azo bond [27]. As a result of the high effect of this system, both bands were disappeared after about 180 min, which due to the fragmentation of azo links and “benzene-like” structures.

**Figure 7.**
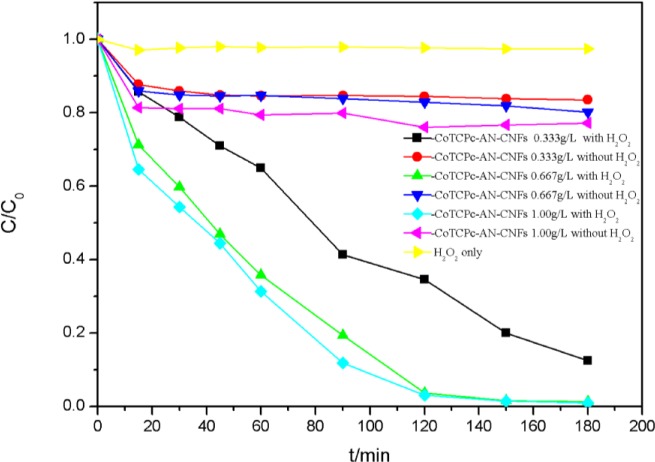
Concentration change of CoTCPc-AN-CNFs in the decolorization [*C*(AO7) = 5.00 × 10^−5^ mol/L, *C*(H_2_O_2_) = 4.85 × 10^−2^ mol/L, 25 °C].

**Figure 8.**
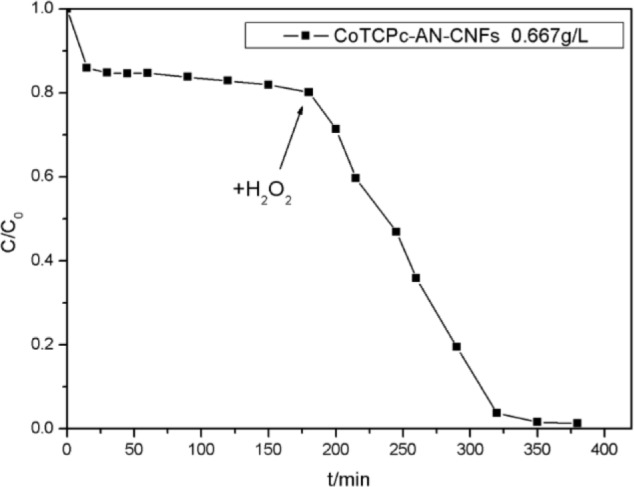
Addition of H_2_O_2_ after dynamic equilibrium [*C*(AO7) = 5.00 × 10^−5^ mol/L, *C*(H_2_O_2_) = 4.85 × 10^−2^ mol/L, 25 °C].

Figure 7 illustrates decolorization of AO7 at different catalyst concentrations. In the presence of CoTCPc-AN-CNFs, the concentration of AO7 was reduced within 20 min and was almost constant during the subsequent 160 min, which indicated the absorption process had reached dynamic equilibrium at 25 °C. About 20% of AO7 was absorbed onto CoTCPc-AN-CNFs. However, in the coexistence of CoTCPc-AN-CNFs and H_2_O_2_, the concentration of AO7 declined rapidly and complete decoloration was achieved in 180 min (Figure 7). The decolorization rate and decolorization efficiency increased with catalyst concentration. The curve of decolorization rate at various catalyst concentrations in the absence of H_2_O_2_ illustrated that the adsorption process reached equilibrium 20 min. When the concentration of CoTCPc-AN-CNFs increased from 0.333 to 1.000 g/L in the absence of H_2_O_2_, the decolorization of AO7 showed an insignificant decrease. The concentration of AO7 declined rapidly in the presence of H_2_O_2_ when the concentration increased from 0.333 to 1.00 g/L. From the Figure 5, the optimum concentration of CoTCPc-AN-CNFs was 0.667 g/L. As described before [8,9], owing to the similar properties between the MWCNT and the CNFs, the decoloration of AO7 in CoTCPc-AN-CNFs/H_2_O_2_ system involved two processes: adsorption of AO7 onto the CoTCPc-AN-CNFs and then immediate catalytic oxidation of the absorbed AO7. We further investigated whether catalytic oxidation of AO7 could still occur after the absorption reached equilibrium. As shown in Figure 8, H_2_O_2_ was added after dynamic equilibrium in the moment of 200 min, catalytic oxidation of AO7 still occurred like before. This result indicated that the absorbed AO7 could be oxidized quickly after the addition of H_2_O_2_ and the consumption of absorbed AO7 was replenished continuously from AO7 in aqueous solution until the decoloration was complete.

**Figure 9.**
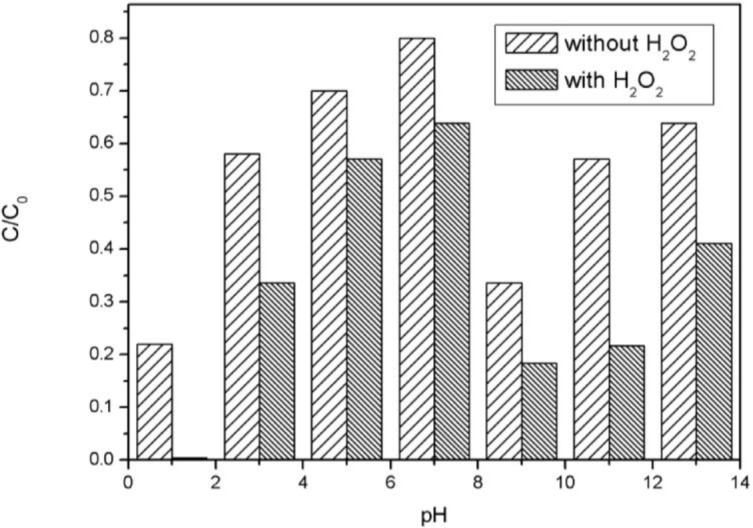
Effect of pH on catalytic oxidation of AO7 [*C*(AO7) = 5.00 × 10^−5^ mol/L, *C*(H_2_O_2_) = 4.85 × 10^−2^ mol/L, *C*(CoTCPcNa-AN-CNFs) = 0.667 g/L, 25 °C] after 1 h.

The catalytic reaction with MPcs depends on electron donating and accepting between the coordination central metal ions and reactants which are sensitive to pH. The catalytic oxidation and absorption process of AO7 with CoTCPc-AN-CNFs were under different pH conditions studied as shown in Figure 9. The absorption and decolorization efficiency of CoTCPc-AN-CNFs increased with the decrease of initial pH and reached the highest at pH = 1. About 98% decolorization and 78% absorption were observed at pH = 1. This is in agreement with that the coordination between the dye and catalyst is more efficient in acidic conditions for the acidic groups in AO7 and CoTCPc-AN-CNFs. AO7 has a sulfuric group, is negatively charged in alkaline conditions, which causes the lower absorbance of dyes. On the other hand, more efficient formation of hydroxyl radicals from hydrogen peroxide occurs in alkaline conditions. Resulting, the optimum pH value for this catalytic system is 9, and 80% decolorization of AO7 was observed.

**Figure 10.**
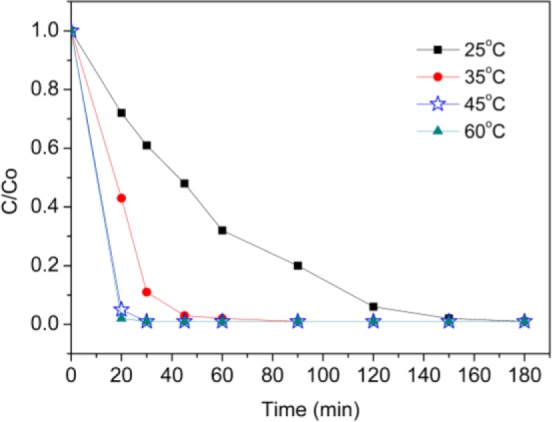
Effect of temperature on catalytic oxidation of AO7 [*C*(AO7) = 5× 10^−5^ mol/L, *C*(H_2_O_2_) = 4.85 × 10^−2^ mol/L, *C*(CoTCPcNa-AN-CNFs) = 0.667 g/L, 25 °C].

Similarly, the optimum temperature of this catalyst system was determined by evaluating the dye decolorization at different temperatures (Figure 10). It was found that both of decolorization rate and decolorization efficiency increased when raise the temperature. Room temperature has been chosen for considering energy conservation and environmentally friendly.

**Figure 11.**
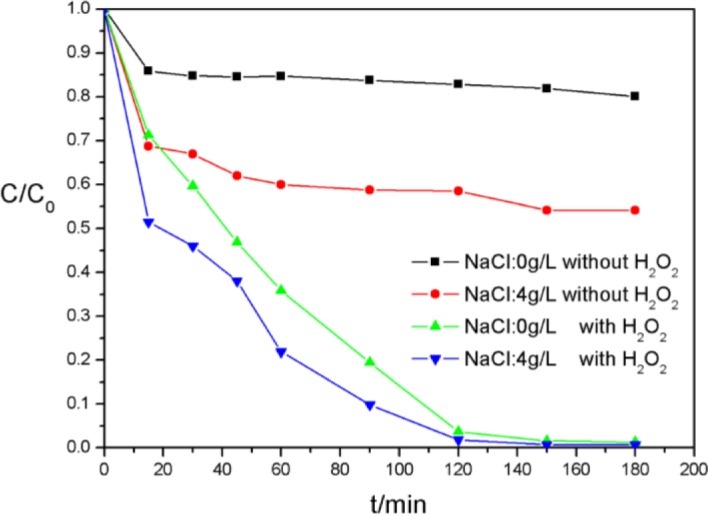
Effect of salinity on catalytic oxidation of AO7 [*C*(AO7) = 5 × 10^−5^ mol/L, *C*(H_2_O_2_) = 4.85 × 10^−2^ mol/L, *C*(CoTCPcNa-AN-CNFs) = 0.667 g/L, 25 °C].

Inorganic salts are prevailing accelerant widely used in the textile dyeing industry for accelerating dye transfer from aqueous solution to fiber phase, such as NaCl, Na_2_SO_4_ and so on. NaCl has been chosen to evaluate the dye decolorization of AO7 (Figure 11). The addition of NaCl caused 10% absorption increasing on CoTCPcNa-AN-CNFs without H_2_O_2_ and 10% decolorization enhancing on CoTCPcNa-AN-CNFs with H_2_O_2_ in the beginning. Unfortunately, the average rate of decolorization was not increased in the presence of NaCl. As we described [19], the existence of Cl^−^ could not prevent the decolorization of AO7, which usually happened in a free hydroxyl radicals participatory reaction. Moreover, the presence of Na^+^ was identified as increases the Zeta (ζ) electric potential which makes the transfer of these electronegative dyes more easily from aqueous phase to solid phase, thus facilitating the catalytic oxidation.

**Figure 12.**
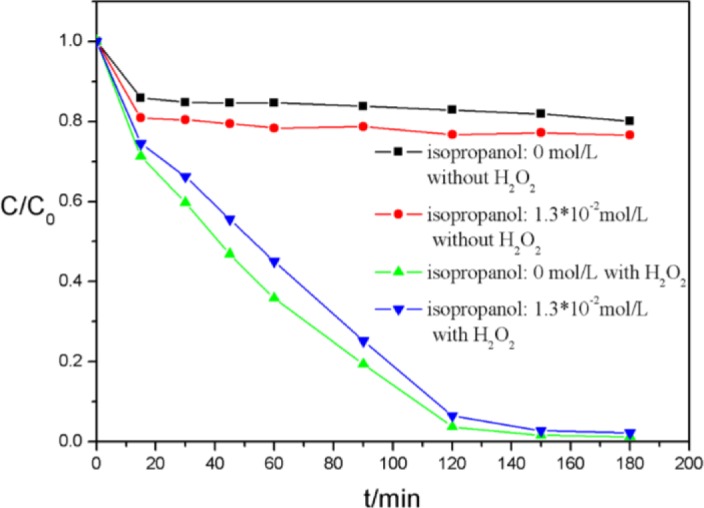
Effect of isopropanol on catalytic oxidation of AO7 [*C*(AO7) = 5 × 10^−5^ mol/L, *C*(H_2_O_2_) = 4.85 × 10^−2^ mol/L, *C*(CoTCPcNa-AN-CNFs) = 0.667 g/L, 25 °C].

Free hydroxyl radicals (such as ·OOH) have a key role in the MPcs and H_2_O_2_ catalytic system [19]. In order to evaluate the effect of these radicals, isopropanol, which is usually used as a quencher, was employed to scavenge the active species, as shown in Figure 12. No obvious changes were observed in both absorption and decolorization of AO7, which implied that ROSs (reactive oxygen species) such as ·OH or ·OOH, played comparatively insignificant roles in AO7 degradation [28]. Therefore, we believe that the introduction of CNFs resulting a new reaction mechanism through non-radical pathways. In contrast to our earlier study [21], the introduction of benzene ring between CNFs and MPcs could not weaken the electronic properties of CNFs.

## 3. Experimental Section

### 3.1. Materials and Reagents

Carbon nanofibers (CNFs) were supplied by Tokyo Chemical Industry Co., Ltd. (Tokyo, Japan). Acid Orange 7 (AO7) and *p*-iodoaniline were obtained from J&K Chemical Company (Shanghai, China) and used without further purification. O-(7-Azabenzotriazol1yl)*N*,*N*,*N*’,*N*’-tetramethyluronium hexafluophosphate (HBTU), *N*-Hydroxybenzotrizole (HOBt), Ethyldiisopropylamine (DIEA) were obtained from J&K Chemical Company and used without further purification. Hydrogen peroxide (9.7M, Sinopharm Chemical Reagent Co., Ltd., Shanghai, China) was of reagent grade. Doubly distilled water was used throughout. *N*,*N*-dimethylformamide (DMF, Sinopharm Chemical Reagent Co., Ltd., Shanghai, China), acetone, sodium chloride and other regents were of analytical reagent.

### 3.2. Preparation of CoTCPcNa-AN-CNFs

Cobalt tetracarboxylphthalocyanine (CoTCPc) was synthesized from 4-carboxylphthalic acid, cobalt chloride hexahydrate and urea as described [26]. The raw CNFs were first immersed in a 6 N HCl and sonicated for 4 h to remove any metal catalyst particles. The precipitate was filtered through 0.22 μm membranes, and then rinsed with distilled water in order to obtain purified CNFs. Finally, the black product was dried at 70 °C in a vacuum. 4-aminophenyl carbon nanofibers (AN-CNFs) were prepared according to the method reported by James M. Tour and co-worker [25] with some modification. Briefly, CNFs (0.80 g) were added to a flame-dried 250 mL three-necked flask fitted with a cold finger condenser and stirrer. NH_3_ (200 mL) was condensed into the flask using a dry ice and acetone bath. Once the NH_3_ was collected, the solution was homogenized using an adjustable magnetic stirrer. Sodium metal (5.00 g) was added to the ammonia solution, which remained a dark blue color indicating an excess of sodium had been added and all possible sites on the CNFs had been activated. After the solution homogenized for 30 min, *p*-iodoaniline (14.00 g) was added, and homogenization continued for 5 h at −78 °C. Then the homogenization was stopped and the solution was removed from the dry ice acetone bath and allowed to warm to room temperature while the NH_3_ was distilled into a scrubber system. Reaction workup included the successive additions of methanol (80 mL) and water (80 mL). This mixture was then filtered using a 0.22 μm membrane. The 4-aminophenyl carbon nanofibers (AN-CNFs) was dispersed via sonication and successively washed and filtered with acetone, dimethylformamide, acetone, water, and acetone. Each filtration was over a 0.22 μm membrane. Finally, the AN-CNFs were dried at 70 °C in vacuum. AN-CNFs (0.80 g), DMF (100 mL), CoTCPc (0.08 g) were added to a 250 mL three-necked flask successively and then mixed under sonication for 1 h. O-(7-Azabenzotriazol1yl)*N*,*N*,*N*’,*N*’-tetramethyluronium hexafluophosphate (HBTU), *N*-Hydroxybenzotrizole (HOBt), Ethyldiisopropylamine (DIEA) were used as the catalyst system of forming amido bond. The molar ratio of CoTCPc: HBTU: HOBt: DIEA is 1:10:10:20. After all reagents were added completely, the reaction system was heated to 60 °C under N_2_ atmosphere for 48 h. The excess CoTCPc was removed completely by washing with anhydrous DMF until the solution was colorless. The precipitate was then dispersed in saturation NaHCO_3_, and –COOH of CoTCPc, which fixed on CNFs turned to –COONa. This transformation made the catalyst more hydrophilic and enhanced the probability of the interaction between MPcs ring and AO7. The product (CoTCPcNa-AN-CNFs) was dispersed via sonication and successively washed and filtered with water, acetone, water, and acetone. Each filtration was over a 0.22 μm membrane. Finally, the CoTCPcNa-AN-CNFs were dried at 70 °C in vacuum.

### 3.3. Characterization of CoTCPcNa-AN-CNFs

The chemical structures of CNFs, AN-CNFs and CoTCPcNa-AN-CNFs were analyzed with X-ray photoelectron spectroscopy (XPS) measurements (Kratos AXIS Ultra DLD). The standard Mg Kα (1256.6 eV) X-ray source operated at 10 mA and 15 kV. All binding energies were referenced to Au (4f_7/2_) at 84 eV. The thermal stability of CNF, AN-CNF and CoTCPcNa-AN-CNFs was investigated using Mettler-Toledo TGA under a nitrogen atmosphere at a flow rate of 100 mL·min^−1^, with a heating rate of 20 °C·min^−1^. The cobalt content in CoTCPcNa-AN-CNFs was measured by atomic absorption spectrometry (Thermo Sollar M6), allowing calculation of the content of CoTCPcNa in CoTCPcNa-AN-CNFs. The mass content of CoTCPcNa in CoTCPcNa-AN-CNFs is 2.67% (m/m). The morphology of CNFs, AN-CNFs and CoTCPcNa-AN-CNFs were carried out by transmission electron microscope using JEM-2010. Nitrogen adsorption-desorption isotherms were carried out at 77 K using a Micromeritics ASAP 2020 analyzer. Before adsorption, the samples were out-gassed at 323 K for 10 h. Fourier transform infrared spectroscopy (FTIR) spectra of a sample in KBr pellet were recorded on a Nicolet Avatar 370 spectrometer.

### 3.4. Analytical Methods

Catalytic oxidation of AO7 was done at 25 °C using H_2_O_2_ as the oxidant. CoTCPcNa-AN-CNFs dispersed by sonication were added to the reaction solution, and the initial concentration of AO7 was 5 × 10^−5^ mol/L. The pH values were adjusted by addition of dilute aqueous NaOH or HCl. All reactions were carried out in a container agitated with a magnetic stirrer. After reaction, reaction solution was passed through 0.22 μm pore size cellulose filters and analyzed immediately with a UV-Vis spectrometer (Hitachi U-3010, Hitachi, Ltd., Tokyo, Japan) by measuring the removal of AO7 (absorbance at 484 nm) at the maximal wavelength.

## 4. Conclusions

Amine-modified CNFs were obtained through the Billups reaction, and were used as supports of CoTCPc for the catalytic oxidation of AO7 in the CoTCPc-AN-CNFs/H_2_O_2_ system. The introduction of CNFs into the CoTCPc catalyst system leads a synergistic effect between the absorption and the degradation of AO7. Other than reported before, the benzene ring was first introduced between CNFs and MPcs. However, its catalytic efficiency and electronic properties would not weaken according to our research. Besides, many effects were studied to find optimum reaction conditions. Therefore, Amine-modified CNFs present special structural and electronic characteristics, as well as acting as a support of catalysts in our CoTCPc-AN-CNFs/H_2_O_2_ system. Such an interesting function of CNFs may provide a new strategy for the design of highly efficient catalysts.
